# Biomechanical Analysis of Various Reconstructive Methods for the Mandibular Body and Ramus Defect Using a Free Vascularized Fibula Flap

**DOI:** 10.1155/2020/8797493

**Published:** 2020-03-13

**Authors:** Xian Li, Chao Jiang, Hui Gao, Chunjuan Wang, Chao Wang, Ping Ji

**Affiliations:** ^1^Stomatological Hospital of Chongqing Medical University, Chongqing, China; ^2^Chongqing Key Laboratory of Oral Diseases and Biomedical Sciences, Chongqing, China; ^3^Chongqing Municipal Key Laboratory of Oral Biomedical Engineering of Higher Education, Chongqing, China

## Abstract

Several different methods exist for reconstructing the mandibular body and ramus defect with the use of a free vascularized fibula flap, but none have adequately addressed the long-term mechanical stability and osseointegration. The aim of this study is to compare the biomechanics of different surgical methods and to investigate the best approach for reconstructing the mandibular body and ramus defect. Five finite element models based on different reconstructive methods were simulated. Stress, strain, and displacement of connective bone sections were calculated for five models and compared. The models were printed using a 3D printer, and stiffness was measured using an electromechanical universal testing machine. The postoperative follow-up cone beam computed tomography (CBCT) was taken at different time points to analyze bone mineral density of connective bone sections. The results showed that the “double up” (DU) model was the most efficient for reconstructing a mandibular body and ramus defect by comparing the mechanical distribution of three sections under vertical and inclined loading conditions of 100 N. The stiffness detection showed that stiffness in the DU and “double down” (DD) models was higher compared with the “single up” (SU), “single down” (SD), and “distraction osteogenesis” (DO) models. We used the DU model for the surgery, and postoperative follow-up CBCT showed that bone mineral density of each fibular connective section increased gradually with time, plateauing at 12 weeks. We conclude that a free vascularized fibula flap of the DU type was the best approach for the reconstruction of the mandibular body and ramus defect. Preoperative finite element analysis and stiffness testing were shown to be very useful for maxillofacial reconstruction.

## 1. Introduction

Mandibular defects can often be caused by a number of phenomena, including but not limited to tumors, trauma, and inflammation. The reconstruction of a mandibular defect with a vascularized free fibula flap is a well-established method [1]. A vascularized free fibula flap is longer, has long vascular pedicles that help in the mandibular reconstruction [2], and has been shown to be highly reliable and adaptable [3, 4]. However, in addition to early complications such as a vascular crisis, long-term complications can occur between the grafted fibula bone and the host's native mandible [5–7], such as delayed healing or poor union, which compromise long-term stable occlusion and oral rehabilitation. Recent CT evaluations reported that there was about 9% [8] to 20% [9] of nonunion in the mandibular reconstruction. Morphology and biomechanics of the fibula have been thought to play a role in the poor osseointegration of a mandibular reconstruction [10]. Therefore, the type of fibular flap that is applied in surgery plays a critical role in the success of long-term stable osseointegration [11]. Many studies have reported on the use of 3D finite element analysis (FEA) in mandibular reconstruction [12–16]. However, most of these studies deal with a postoperative analysis. In our study, we aimed to compare the biomechanics of different surgical methods preoperatively and to investigate the best approach for reconstructing the mandibular body and ramus defect. Moreover, we further verified the preoperative biomechanical analysis using postoperative bone mineral density (BMD) analysis of fibular connecting sections. Preoperative stress, strain, displacement analysis, and stiffness detection provide guidance for the selection of surgical methods. Postoperative bone mineral density test further verifies the results of preoperative stress, strain, displacement analysis, and stiffness detection. Previous studies have simulated and quantitatively analyzed the stress and strain distribution on a normal mandible under physiological occlusal loadings by 3D finite element models [15]. In this study, we expanded these earlier observations and analyzed the stress, strain, and displacement of connective bone sections using five different bone reconstruction models.

We used the mandibular body and ramus defect as a prototype, since these disorders are commonly encountered in clinical practice [17]. The fibula flap method for mandibular reconstruction was first reported by Hidalgo in 1989 [18], after which Horiuchi et al., in 1995 [19], reported the double-barrel fibular graft for mandibular reconstruction. Single fibula grafts are divided into two types [20]. The first type of single fibula flap, termed “single up” (SU), lies in the direction of the alveolar ridge. This type can achieve a good dental implant, but the lower margin of the mandible has a step. The second type of single fibula flap, termed “single down” (SD), lies in the direction of the lower margin of the mandible. This type can achieve a good outline of the lower margin of the mandible, but it is difficult to achieve a good bite following a dental implant. Due to the disadvantages of a single fibula flap mentioned above, a double-barrel fibula graft was proposed and seems to effectively address these two issues [21]. However, in many patients, the height of a complete double-barrel fibula is greater than the height of the mandible, which will result in an insufficient space for denture repair or early contact [22]. For these patients, a double-barrel fibula graft is divided into two types [23]. In the first type, termed “double up” (DU), the folded section is cut obliquely and fixed in the direction of the alveolar ridge. In the second type, termed “double down” (DD), the folded section is obliquely cut and fixed in the direction of the lower margin of the mandible. In addition to these four types, another type of reconstruction, termed “distraction osteogenesis” (DO), consists of a single fibula flap fixed to the lower margin of the mandible, where the height of the alveolar bone is distracted by oblique distraction osteogenesis to meet the requirement of a dental implant [24]. The five methods mentioned above are all used in current surgical practice [25].

Among these five types of reconstruction, it is not known which method has the best biomechanical characteristics and can thus lay the best foundation for its long-term stable occlusion [26]. In this study, we analyzed the biomechanical characteristics of three connecting sections (S1, S2, and S3) using the five different models under vertical and inclined loading conditions of 100 N and printed the simulation models with a 3D printer to perform a stiffness test. The optimal model was chosen and applied clinically. Postoperative bone mineral density (BMD) of fibular connecting sections were measured at different time points to verify the preoperative biomechanical analysis. Finite element method is an effective tool to evaluate the biomechanical properties of different anatomical structures. In order to further verify the reliability of finite element analysis results, all models were analyzed by the mechanical test and FEA analysis. The results showed that the trend of analysis was consistent with the clinical observation.

## 2. Materials and Methods

### 2.1. Patient Preparation

A 28-year-old female patient was planned to receive a mandibular reconstruction with a free vascularized fibula flap due to a recurrent ameloblastoma of the left mandibular body and ramus at the Department of Oral and Maxillofacial Surgery of the Affiliated Stomatological Hospital of Chongqing Medical University. On CBCT examination, the tumor was found to involve the left mandibular body and ramus, and the leg CTA examination showed no abnormality in the calf artery vascular bundle. The research protocol was approved by the ethics committee of the Affiliated Stomatological Hospital of Chongqing Medical University (reference #2018-7). Full written informed consent was obtained for the chosen surgery method and use of CT images. All procedures performed in this study were in accordance with the ethical standards of the 1964 Helsinki Declaration (http://www.wma.net) and its later amendments.

### 2.2. Finite Element Modeling

**Figure 1:**
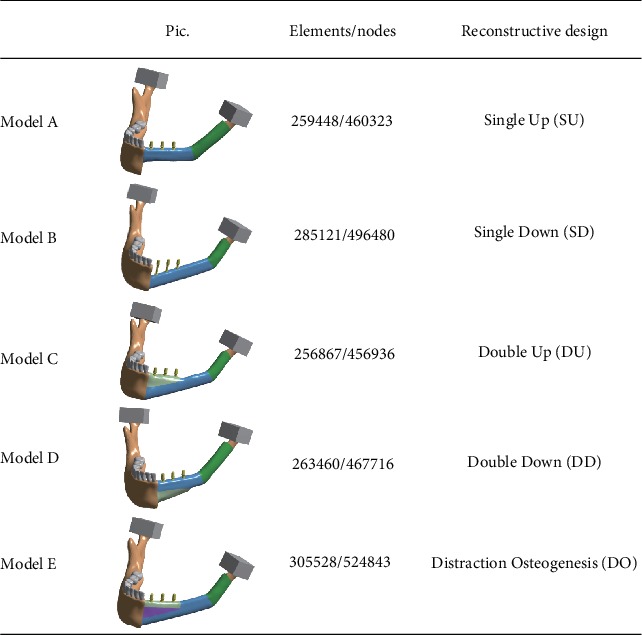
FE models and the number of nodes and elements of FE models.

A 3D model of the mandibular bone was extracted from CBCT images. The CT images consisted of 452 transversal sections with a slice thickness of 0.425 mm and a pixel width of 0.398 mm. The data were imported into Mimics (Materialise, Leuven, Belgium) and Geomagic (Geomagic Company, NC, USA) to generate the geometric model. Five different mandibular reconstruction surgical procedures were developed (Figure 1) [27, 28]. We printed 10 test models for each digital model and obtained 10 sets of measurements.

*Model A (single up, SU)*: two segments of the fibula replaced the mandibular body and the ramus, respectively. A single-layer fibula, lying in the direction of the alveolar ridge, was reconstructed for the mandibular body defect.

*Model B (single down, SD)*: the ramus defect reconstructive design was the same as in Model A. A single-layer fibula, lying in the direction of the lower margin of the mandible, was reconstructed for the mandibular body defect.

*Model C (double up, DU)*: the ramus defect reconstructive design was the same as in Model A. A double-barrel fibula was grafted to reconstruct the mandibular body defect, and the folded section was cut obliquely and fixed in the direction of the alveolar ridge.

*Model D (double down, DD)*: the ramus defect reconstructive design was the same as in Model A. A double-barrel fibula was grafted to reconstruct the mandibular body defect, and the folded section was cut obliquely and fixed in the direction of the lower margin of the mandible.

*Model E (distraction osteogenesis, DO)*: the ramus defect reconstructive design was the same as in Model A. A single-layer fibula was fixed to the lower margin of the mandible, and the height of the alveolar bone was distracted by oblique distraction osteogenesis to meet the requirement of a subsequent dental implant.

Implants were finally implanted into the grafted fibula and were modeled using SolidWorks (SolidWorks Corp., Dassault Systemes Concord, MA, USA). The finite element (FE) models were meshed using 10-node solid tetrahedral elements in ANSYS Workbench (Swanson Analysis Systems Co., Houston, TX, USA) [29]. The stress, strain, and displacement of each node of each connection section of each model were collected using ANSYS software. A global element edge length of 0.5 mm was implemented following a convergence test, which assessed the balance between modeling accuracy and cost. The detailed element assignment is listed in Table 1. The final model includes residual mandible, segmented fibula, 3 dental implants, TMJ discs, periodontal ligaments (PDL), and 9 teeth (32-47). The criteria used for the bone strain values for this FEA were according to a method described previously [30]. In brief, the genetically determined disuse-mode threshold strain range was classified as described elsewhere [30]. The various strain ranges are described below. The 50 microstrain is the strain where the maximal disuse-mode activity occurs and above which it begins to decline or turn off. 1000–1500 microstrain is the strain range whereby the mechanically controlled modeling function of increasing a bone's strength would usually turn on. 3000 microstrain is the bone's microdamage strain threshold range whereby irreversible microdamage can begin to accumulate. To promote bone remodeling, the strain associated with favorable models should be in the range of 50–3000 *με* under any conditions.

**Figure 2:**
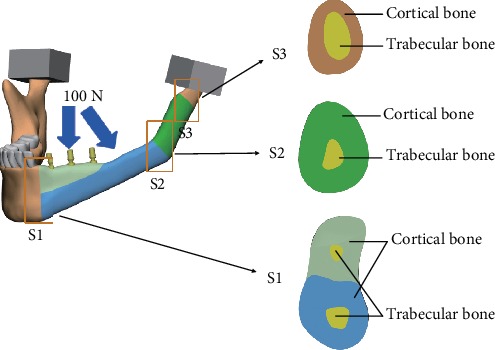
Each model was applied with a vertical and inclined force of 100 N each. The three bone connective sections (S1, S2, and S3) were marked by the golden boxes and amplified.

Two types of masticatory force (vertical 100 N and inclined 45°100 N) were modeled as a concentrated force and applied on the abutment to simulate physiological loading conditions (Figure 2). To simulate the boundary conditions, the top, medial, and distal borders of the condyle process were considered to be in contact with each other. The sliding contact was applied to define the interfaces between the condyle process and the disc in the ANSYS Workbench. Sliding between the interfaces was allowed during the simulation process. Other contact interfaces were bonded (Table 2).

### 2.3. Stiffness Testing

The five models were printed using a 3D printer, and each model was placed on an electromechanical universal testing machine (C44, MedEx industrial systems, China) for compression testing [31, 32]. They were divided into 5 groups according to 5 different models, and 10 measurements were obtained from each group. The stiffness values of each group were assessed from the electromechanical universal testing machine, and these figures were statistically analyzed using one-way analysis of variance (ANOVA) [33].

### 2.4. Postoperative Bone Density Testing

The biomechanical reconstructive method was optimized according to the analysis mentioned above and was subsequently used in the operation. The operation was successfully performed, and the patient recovered well following surgery. CBCT images were obtained at 2 weeks (2w), 12 weeks (12w), and 24 weeks (24w) following surgery [34, 35]. The gray value detection function of the CBCT software was used to detect bone mineral density. We took several points randomly on each bone joint section to extract the gray value of these points and calculated their mean value and standard deviation for comparison using one-way ANOVA.

## 3. Results

### 3.1. Mechanical Distribution

**Figure 3:**
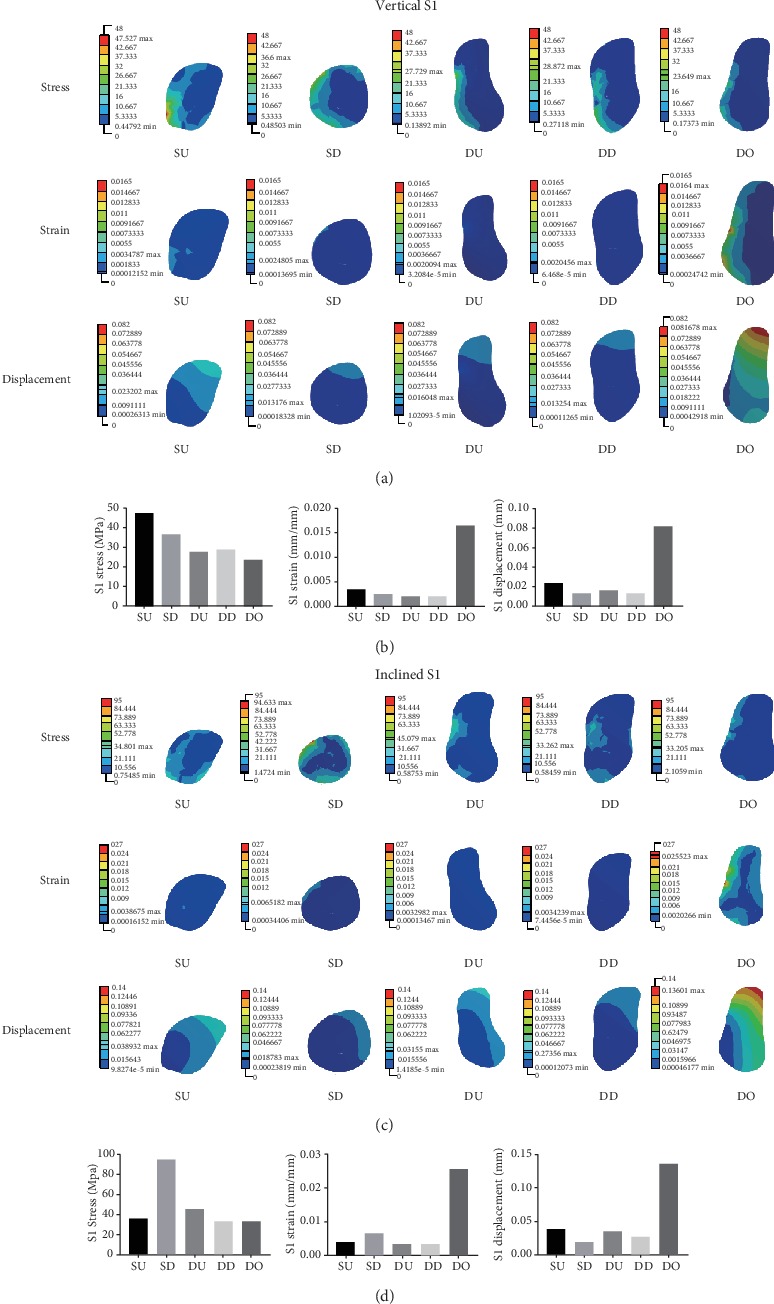
(a, c) The mechanical distribution in S1 in the SU, SD, DU, DD, and DO models, under vertical and inclined loading conditions of 100 N, respectively. (b, d) Histograms comparing the maximum values of stress, strain, and displacement in the five models under vertical and inclined loading conditions of 100 N, respectively.

**Figure 4:**
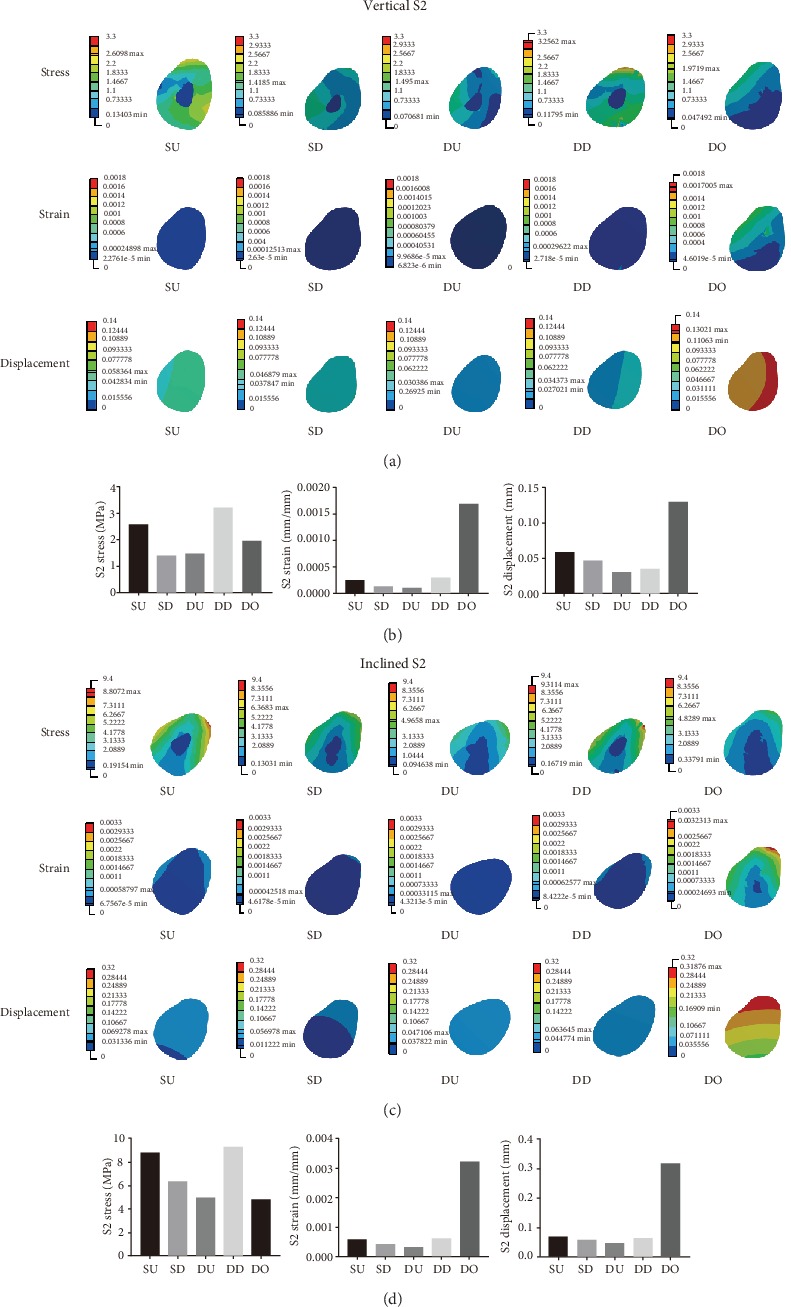
(a, c) The mechanical distribution in S2 in the SU, SD, DU, DD, and DO models, under vertical and inclined loading conditions of 100 N each. (b, d) Histograms comparing the maximum values of stress, strain, and displacement in S2 in the five models under vertical and inclined loading conditions of 100 N, respectively.

**Figure 5:**
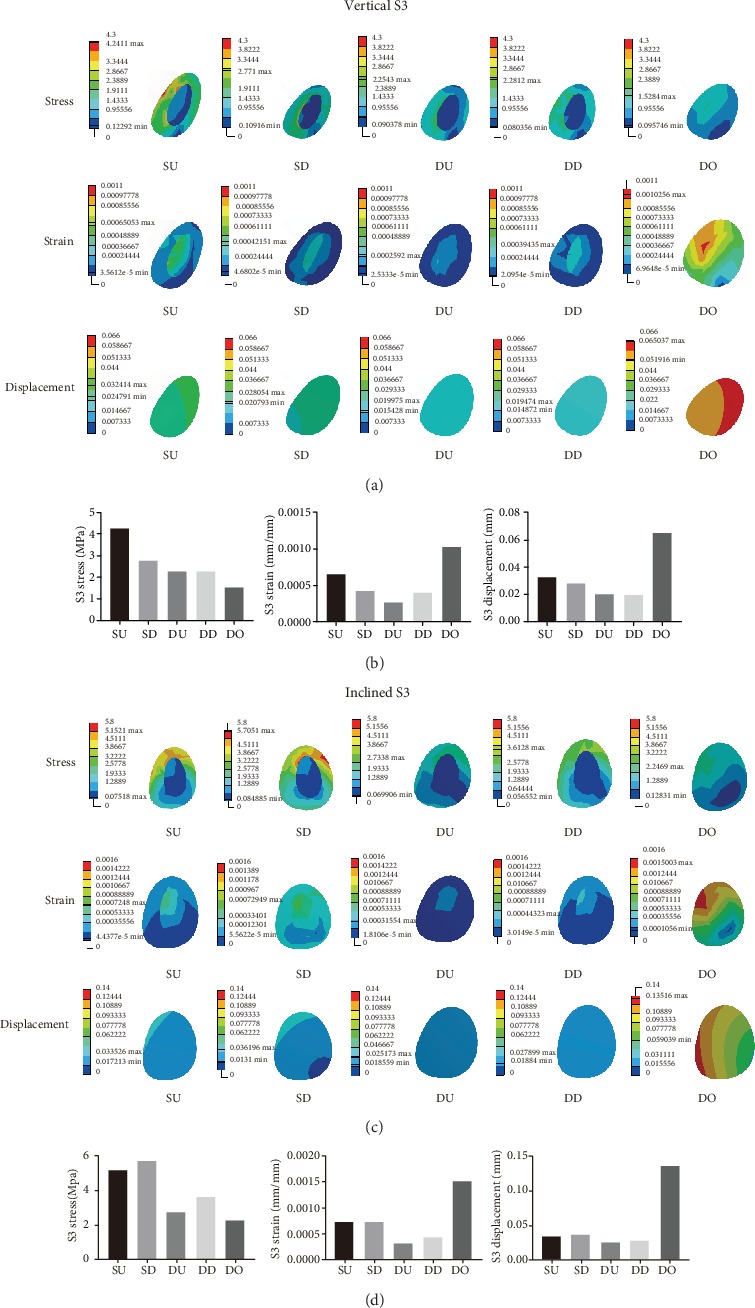
(a, c) The mechanical distribution in S3 in the SU, SD, DU, DD, and DO models under vertical and inclined loading conditions of 100 N, respectively. (b, d) Histograms comparing the maximum values of stress, strain, and displacement in S3 in the five models under vertical and inclined loading conditions of 100 N, respectively.

Under vertical and inclined loading conditions of 100 N each, the mechanical distributions of three sections (S1, S2, and S3) in Models A-E were collected and compared. The mechanical distributions (stress, strain, and displacement) of S1, S2, and S3 in the five models are shown in Figures 3–5.

Figures 3(a) and 3(c) show the detailed mechanical distribution of stress, strain, and displacement in S1 in the five models under vertical and inclined loading conditions of 100 N each. Figures 3(b) and 3(d) show the histograms of the stress, strain, and displacement distribution in S1 under vertical and inclined loading conditions of 100 N each. When analyzing S1, under vertical loading conditions of 100 N, the largest value of the maximum of stress, strain, and displacement was found for SU, DO, and DO, respectively. The smallest value of the maximum of stress, strain, and displacement was observed for DO, DU, and SD, respectively. Under inclined loading conditions of 100 N, the largest value of the maximum of stress, strain, and displacement was found for SD, DO, and DO, respectively. The smallest value of the maximum of stress, strain, and displacement was found for DO, DU, and SD, respectively.

Figures 4(a) and 4(c) show the detailed mechanical distributions of stress, strain, and displacement in S2 in the five models under vertical and inclined loading conditions of 100 N each. Figures 4(b) and 4(d) show the histograms of stress, strain, and displacement distributions in S2 under vertical and inclined loading conditions of 100 N each. When analyzing S2, under vertical loading conditions of 100 N, the largest value of the maximum of stress, strain, and displacement was found for DD, DO, and DO, respectively, and the smallest value of the maximum stress, strain, and displacement was observed for SD, DU, and DU, respectively. Under inclined loading conditions of 100 N, the largest value of the maximum of stress, strain, and displacement was seen in DD, DO, and DO, respectively, and the smallest value of the maximum of stress, strain, and displacement was obtained in DO, DU, and DU, respectively.

Figures 5(a) and 5(c) show the detailed mechanical distribution of stress, strain, and displacement in S3 in the five models under vertical and inclined loading conditions of 100 N each. Figures 5(b) and 5(d) show the histograms of the stress, strain, and displacement distribution in S3 under vertical and inclined loading conditions of 100 N each. When analyzing S3, under vertical loading conditions of 100 N, the largest value of the maximum of stress, strain, and displacement was found for SU, DO, and DO, respectively. The smallest value of the maximum of stress, strain, and displacements was seen for DO, DU, and DD, respectively. Under inclined loading conditions of 100 N, the largest value of the maximum of stress, strain, and displacement was observed for SD, DO, and DO, respectively, and the smallest value of the maximum of stress, strain, and displacement was seen in DO, DU, and DU, respectively.

Collectively, the SU model obtained 0 minimums and 2 maximums, the SD model obtained 3 minimums and 2 maximums, the DU model obtained 9 minimums and 0 maximums, the DD model obtained 1 minimum and 2 maximums, and the DO model obtained 5 minimums and 12 maximums. The higher the number of minimums and the lower the number of maximums, the better the model. According to the results of the mechanical distributions of the three investigated sections (S1, S2, and S3) under vertical and inclined loading conditions of 100 N, the DU model got the highest number of minimums and had no maximum. Compared with the other models, the value of strain associated with the DU model was almost in the range of 50–3000 *με* under different conditions [30]. Therefore, the DU model was the ideal choice for reconstructing the mandibular body and ramus defect in this particular case.

### 3.2. Median Values of Mechanical Distributions

**Figure 6:**
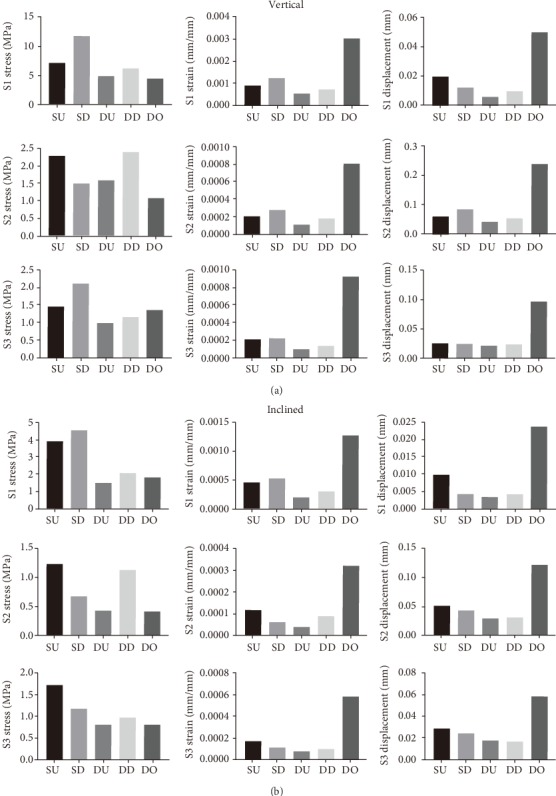
Medians of vertical and inclined forces in S1, S2, and S3. (a) Medians of stress, strain, and displacement values of S1, S2, and S3 in the SU, SD, DU, DD, and DO models under vertical loading conditions of 100 N. (b) Medians of stress, strain, displacement values of S1, S2, and S3 in the SU, SD, DU, DD, and DO models under inclined loading conditions of 100 N.

The data of all finite element nodes of S1, S2, and S3 in each model under vertical and inclined loading were analyzed, and since they were not normally distributed, the medians of the data in each group were compared using histograms (Figure 6).

Analysis of the medians of the mechanical distributions of three sections (S1, S2, and S3) under vertical and inclined loading conditions of 100 N in the five different models showed that the DU model had the smallest medians of strain and displacement, even though its median stress values were not the smallest. In this respect, the DU model was the best choice for reconstructing the mandibular body and ramus defect in this particular case.

### 3.3. Result of Stiffness Testing

**Figure 7:**
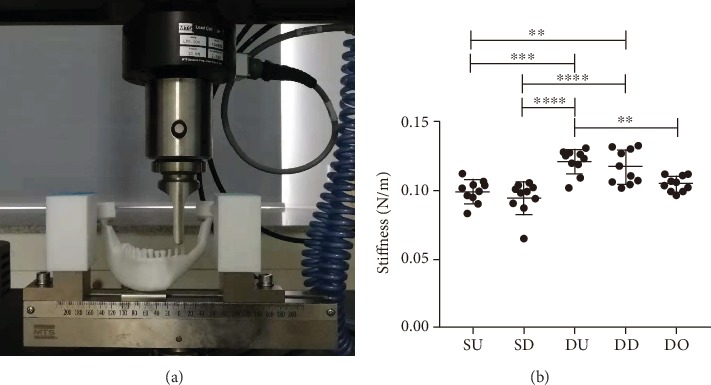
Stiffness detection and statistical analysis of all models. (a) Five reconstructive models were printed out, and stiffness was measured using an electromechanical universal testing machine. (b) Scatterplot comparison of stiffness measurements. Statistical analysis: one-way ANOVA (^∗^*p* < 0.05, ^∗∗^*p* < 0.01, ^∗∗∗^*p* < 0.005, ^∗∗∗∗^*p* < 0.001).

To further assess the best method for reconstructing the mandibular body and ramus defect, the five reconstructive models were printed using a 3D printer and the stiffness was measured using an electromechanical universal testing machine. The results showed that the stiffness values in the DU and DD models were higher compared with the SU, SD, and DO models (Figure 7). However, there was no statistically significant difference between stiffness values between the DU and DD models.

### 3.4. Result of Postoperative Bone Density Testing

**Figure 8:**
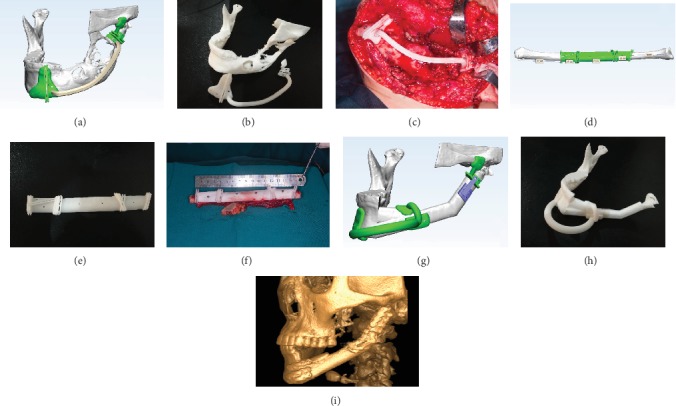
Digital design and operation. (a) Digital design of mandibular tumor resection. (b) 3D printed osteotomy guided plates of the mandible. (c) Intraoperative resection of mandibular tumor resection. (d) Digital design of fibular resection. (e) 3D printed osteotomy guided plate of the fibula. (f) Intraoperative resection of fibular resection. (g) Digital design of fibular segments' emplacement. (h) 3D printed fibular emplacement guided plates. (i) Postoperative CBCT of fibular reconstruction.

**Figure 9:**
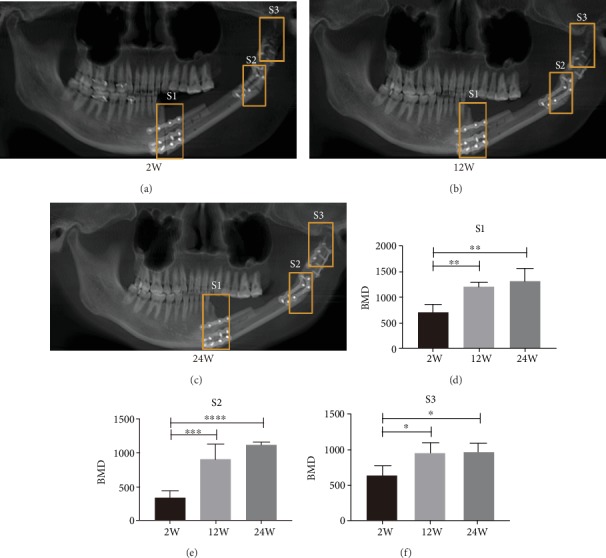
Postoperative CBCT and bone mineral density statistics in connecting sections. (a–c) Postoperative follow-up CBCT at 2w, 12w, and 24w. (d–f) Histogram comparison of bone mineral density of each fibular connecting section (S1, S2, and S3) at 2w, 12w, and 24w. Statistical analysis: one-way ANOVA (^∗^*p* < 0.05, ^∗∗^*p* < 0.01, ^∗∗∗^*p* < 0.005, ^∗∗∗∗^*p* < 0.001).

Based on preoperative finite element modeling analysis and stiffness tests, we decided that the “double up” DU model consisting of a double-barrel of a vascularized free fibula flap graft was the most efficient approach for the reconstruction of the mandibular body and ramus defect and was thus used during the surgery (Figure 8). The postoperative follow-up CBCT was taken at 2w, 12w, and 24w to assess the extent of osseointegration. We found that the gray value of bone mineral density of each fibular connecting section increased gradually with time. The gray values of bone mineral density at 12w and 24w were higher compared with that at 2w. However, no significant difference was found between 12w and 24w (Figure 9).

## 4. Discussion

In this study, we describe the use of a preoperative biomechanical force distribution analysis of three fibular connecting sections (S1, S2, and S3) under vertical and inclined loading conditions of 100 N and show that the DU model was the best approach for reconstructing the mandibular body and ramus defect in this particular case. After the models were printed, stiffness detection showed that the DU and DD models were stiffer compared to the SU, SD, and DO models. After using the DU model in the surgery of our patient, we observed that the bone mineral density of each fibular connecting section gradually increased with time, plateauing at 12w. This early osseointegration was more rapid than reported in an earlier paper [7].

We chose to perform a biomechanical evaluation of the five methods which are currently often used in the clinical practice of mandibular reconstruction, prior to performing surgery in our patient. For the surgery using the DU and DD models, an obliquely cut fibula is folded [36] and the periosteum between two fibular barrels is peeled off to expose the bone surface, and multiple holes are drilled on the bone surface to promote subsequent osseointegration [23]. Since vertical and inclined forces are the two most frequent forces applied on mandibles during daily occlusion and mastication, we chose to simulate this in each model by applying vertical and inclined forces of 100 N each to the fibula's molar area. In terms of long-term stability of the grafted fibula, the fibular connections were the weakest and the most easily concentrated areas, so we chose three fibular connecting sections of each model to measure their stress, strain, and displacement.

For each fibular connecting section of each model, all data of finite element nodes under different loading conditions were obtained. Since these data were nonnormally distributed, we extracted their median and compared them by analysis of histograms. At the same time, we also presented the stress, strain, and displacement histograms of each model's fibular connections under different loading conditions, which allowed an overall analysis of all forces, including the maximum and minimum values.

Nowadays, the mechanical analysis of mandibular reconstruction with a vascularized free fibular flap mainly focuses on certain key methods of mandible reconstruction [37]. However, little is known concerning the type of mechanical distribution induced by different reconstruction methods, especially on the connections between bone segments. It has not yet been reported which method has the best mechanical distribution. Based on the current literature, there is a lack of preoperative biomechanical analysis to guide the selection of mandibular reconstruction. Even if there is a biomechanical analysis for mandibular reconstruction, most of them deal with a postoperative analysis, and the surgical method used, although this might not have been the most optimal one. The best reconstruction uses a method that has long-term stability without placing excessive burden on the connections between bone segments. To assess this, we used a FEA to compare the mechanical distributions of five different types of fibular flap grafting methods. In terms of the maximum of stress, strain, and displacement in S1 under vertical loadings of 100 N, the SU and SD models were subjected to the most stress and the DO model was subjected to the most strain and displacement. Simultaneously, from the perspective of stress, strain, and displacement in S2 and S3 under inclined loadings of 100 N, the SU, SD, and DD models were subjected to the most stress, while the DO model was subjected to the most strain and displacement. According the above analysis, the DU model superseded the SU, SD, DD, and DO models.

In terms of medians of stress, strain, and displacement in S1, S2, and S3 under 100 N vertical and inclined loadings, the DU model obtained the smallest medians of strain and displacement of three sections under the two loading conditions, even though its stress medians were not the smallest. This means that although the DU model was not subjected to the least amount of stress, it did show the smallest strain and displacement, which is preferable to having the smallest stress.

However, since these results were only based on mathematical simulations, we also printed each of the 5 models using a 3D printer and placed them on an electromechanical universal testing machine for a compression test to assess the stiffness of each model [38, 39]. The results showed that the DU and DD models were stiffer than the other three models and that there was no significant difference between the DU and DD models. This result was relatively consistent with the results of the above FEA.

Therefore, in combined computer simulation and stiffness tests, we finally selected the DU model as the best surgical method and completed the operation with the help of our digital designs, 3D printed guided plates [40] and temporomandibular joint positioning [41]. The patient was hospitalized for 2 weeks and underwent a smooth recovery. The CBCT was obtained to assess recovery of the connections of the grafted fibula at 2w, 12w, and 24w after surgery. The bone mineral density of S1, S2, and S3 increased gradually over time. However, there were no statistical differences between 12w and 24w, indicating that the bone mineral density of connections plateaued at 12w. In addition, this method allowed for faster healing than other previously reported reconstruction methods [7]. It is possible to perform dental implants earlier to reconstruct the occlusion, but the earlier recovery of bone mineral density in connections is also dependent on the accuracy of the digital design.

Our study has a few limitations which we would like to mention. First, even though the stiffness and postoperative BMD tests were applied to verify previous analysis results, FEA and stiffness tests might not be able to completely simulate the clinical situation. Second, since vertical and inclined forces reflect the two most important forces applied during mastication, we only simulated these forces. However, there are other forces applied inherent to the movement of the mandibular process which have not been modeled. In addition, reattachment of the masticatory muscles such as the masseter, medial pterygoid, temporalis, and mylohyoid was not fully taken into account in our models. Therefore, further research regarding 3D FEA combined with experimental studies and long-term clinical evaluation is required to validate our approach.

Within the limitations of our study, based on preoperative finite element modeling analysis, stiffness tests, and postoperative follow-up BMD tests, we show that the DU model consisting of a double-barrel of a vascularized free fibula flap graft was the most efficient approach for the reconstruction of the mandibular body and ramus defect. In the future, a biomechanical analysis will need to be conducted before reconstruction of all maxillofacial defects, and the optimal stress model should be selected before the surgery is done. This method provides a biomechanical basis for the choice of the surgical approach in a given patient and can also be applied to other maxillofacial bone defect reconstructions. In the future, not only fibula, iliac crest, and shoulder blade transplantation but also artificial material implantation and biomechanical optimization may be a good direction for further research in this area.

## Figures and Tables

**Table 1:** Material properties used in the FE models.

Material	Young's modulus (GPa)	Poisson's ratio	Reference
Cortical bone	15	0.3	Sarrafpour et al. [42]
Trabecular bone (mandible)	1.5	0.3	Sarrafpour et al.
Trabecular bone (fibula)	0.7	0.3	Park et al. [43]
Teeth	20	0.3	Yu et al. [44]
PDL	0.012	0.45	Sarrafpour et al.
Implant	110	0.3	Bhering et al. [45]
Abutment	110	0.3	Bhering et al.
TMJ disc	0.044	0.4	Sarrafpour et al.

**Table 2:** Contact types set in FE models.

Contact bodies		Contact type
Fibula	Residual mandible	Bonded
Fibula	Fibula	Bonded
Fibula	Implant	Bonded
Abutment	Implant	Bonded
Teeth	PDL	Bonded
Condyle	TMJ disc	Sliding
TMJ disc	Mandibular fossa	Sliding

## Data Availability

The data used to support the findings of this study are included within the article.
